# The Perception of Similarity, Difference and Opposition

**DOI:** 10.3390/jintelligence11090172

**Published:** 2023-08-24

**Authors:** Ivana Bianchi, Roberto Burro

**Affiliations:** 1 Department of Humanities, University of Macerata, 62100 Macerata, Italy; 2 Department of Human Sciences, University of Verona, 37129 Verona, Italy; roberto.burro@univr.it

**Keywords:** same–different, opposition, diversity, similarity, perceptually grounded relationships

## Abstract

After considering the pervasiveness of same/different relationships in Psychology and the experimental evidence of their perceptual foundation in Psychophysics and Infant and Comparative Psychology, this paper develops its main argument. Similarity and diversity do not complete the panorama since opposition constitutes a third relationship which is distinct from the other two. There is evidence of this in the previous literature investigating the perceptual basis of opposition and in the results of the two new studies presented in this paper. In these studies, the participants were asked to indicate to what extent pairs of simple bi-dimensional figures appeared to be similar, different or opposite to each other. A rating task was used in Study 1 and a pair comparison task was used in Study 2. Three main results consistently emerged: Firstly, opposition is distinct from similarity and difference which, conversely, are in a strictly inverse relationship. Secondly, opposition is specifically linked to something which points in an allocentrically opposite direction. Thirdly, alterations to the shape of an object are usually associated with the perception of diversity rather than opposition. The implications of a shift from a dyadic (same/different) to a triadic (similar/different/opposite) paradigm are discussed in the final section.

## 1. Introduction

Sameness, similarity and difference are basic relationships in human perception and cognition. The recognition of these relationships represents the premise for categorization and is therefore the bedrock of language and conceptualization (Addyman and Mareschal 2010; Carstensen and Frank 2021; Goodwin and Johnson-Laird 2005; Halford et al. 2010; Murphy 2002; Tversky 1977). Human talent for relational representation has been seen by many psychologists as being the key to higher-order cognition (e.g., Gentner 2003, 2010; Gentner et al. 2001; Goldwater and Schalk 2016; Mareschal et al. 2010; Penn et al. 2008; Richland and Simms 2015; Wasserman and Young 2010).

In research in the field of Psychology, the widespread use of same–different tasks and those involving a judgment of similarity or diversity reflects the importance of these relationships and the apparent ease with which human beings recognize whether two stimuli (either sensory or related to meanings and concepts) are the same, similar or different. This paper aims to stimulate new considerations concerning the perceptual foundation of these relationships. It also aims to add new data supporting the hypothesis that opposition is a specific perceptual relationship which is distinct from similarity and diversity (a hypothesis which has already been put forward in a small set of studies, reviewed in Section 1.2).

In the introductory section, we show that while sameness and difference (Section 1.1), and similarity and difference (Section 1.2), have each been approached as basic perceptual and conceptual relationships since the very beginning of Experimental Psychology, the study of opposition has been almost exclusively developed within language studies; as such, it has been addressed as a semantic relationship (Section 1.3). This has, until relatively recently, been the mainstream approach. In Section 1.3, however, we refer to a number of studies that have investigated the perceptual characteristics of certain configurations which are specifically associated by adult observers with opposition rather than similarity or diversity. We then present the two studies which were carried out with the aim of contributing to this latter line of research. In the first study (Section 2), the participants were shown pairs of simple bidimensional shapes (a standard one on the left and a comparison shape on the right), and they were asked to rate the extent to which they perceived these pairs of shapes to be similar, different or opposite to each other. The results of this study contribute new information regarding the structure of those visual stimuli which are associated with the perception of each of these three relationships. This, in turn, allowed us to investigate the mutual relationships between them. In a second study (Section 3), we tested the generalizability of the main conclusions resulting from Study 1 using a different task (i.e., a pair comparison task rather than a rating task). In the final discussion (Section 4), the implications of the findings are discussed in relation to current research topics and methodologies and in terms of their support to an approach which focuses on exploring the perceptual foundations of cognition.

### 1.1. Same–Different

The same–different paradigm has been used in many domains of research in the field of Psychology. Its application in contexts in which the perceptual foundation of this relationship is assumed and/or tested are of particular interest in this paper.

A key concept in Psychophysics concerns the “point of subjective equality/inequality”. This refers to the stimulus value at which an observer perceives two stimuli to be subjectively either identical (i.e., same) or slightly distinguishable (i.e., different) from each other along a particular sensory dimension. The point of subjective equality/inequality is often assessed by means of psychophysical methods such as the Method of Adjustment, the Method of Limits or the Method of Constant Stimuli (Kingdom and Prins 2016; Gescheider 2013). All of these methods involve pairs of stimuli that differ in some attribute such as brightness, loudness, duration and so on. Participants are asked to adjust or compare the stimuli until they perceive them as equal or slightly different. The Signal Detection Theory (Macmillan and Creelman 2005; Green and Swets 1966) provides a way of analyzing the participant’s responses in same–different tasks by considering two key factors: sensitivity (d′) and decision criterion (c). Sensitivity represents the ability to discriminate between stimuli which are the same or different, while the decision criterion represents the participant’s bias or willingness to respond “same” or “different” based on the evidence available.

Same–different tasks have been extensively used to test the primitive foundations of abstract representation in Comparative Psychology studies using the match-to-sample and the relational match-to-sample paradigms (for a review, see Carstensen and Frank 2021). In match-to-sample tasks, participants learn to match a cue (e.g., a red dot) with an identical target rather than with a distractor (e.g., a blue circle). In relational match-to-sample tasks, participants are cued with a pair of stimuli that exhibit a given relationship (e.g., sameness, i.e., AA) and are then asked to match these with a target pair that exhibits an identical relationship (i.e., BB, not CD). These methods have frequently been used to test the perception and conceptualization of sameness and difference in non-human animals (for an overview, see Cook and Qadri 2021; Diaz et al. 2021; Lazareva and Wasserman 2017; Scagel and Mercado 2023; Wasserman et al. 2017). Based on the results of these studies, it is acknowledged that many species of animals are capable of learning concepts that presuppose detecting and classifying sameness and difference; examples of these animals include bottlenose dolphins (e.g., Mercado et al. 2000), sea lions (e.g., Kastak and Schusterman 1994), parrots (e.g., Pepperberg 1987), primates (e.g., Wright and Katz 2006), pigeons (e.g., Cook and Brooks 2009), dogs (Scagel and Mercado 2023), bumblebees (Brown and Sayde 2013) and honey bees (Giurfa 2021). This suggests that the ability to detect sameness and difference, and also the ability to transfer this abstract concept from training stimuli to novel stimuli, are not dependent on language competence. It would seem that sameness and difference are two natural concepts that do not require learning (Zentall 2021) and might have evolved in so many animal species due to shared ecological pressure (Katz and Wright 2021; Wasserman et al. 2017; Wright et al. 2003).

Similar conclusions have emerged from investigations into the performance of human infants (for a review, see Hochmann et al. 2016, 2021). Same–different recognition and generalization have been attested as being established in the first year of life (for a review, see Hespos et al. 2020, 2021). Using the preferential looking paradigm and the habituation/dishabituation paradigm, it has been shown that by the age of 7 months, infants manifest a novelty response when comparing an identical pair that they have been shown (i.e., AA) with a new pair (BC). Specifically, they look longer at the novel pair than the familiar pair (Tyrrell et al. 1991; Ferry et al. 2015). If the habituation phase is extended to a series of pairs (e.g., four pairs Ferry et al. 2015 with 7-month-old infants; up to 19 pairs in Addyman and Mareschal 2010, with 8-month-old infants and two alternating pairs in Anderson et al. 2018, with 3-month-old infants), infants are able to transfer these relationships to objects they have not previously seen (Addyman and Mareschal 2010; Ferry et al. 2015; Hespos et al. 2020). The same ability does not seem to be present if the abstraction is based on only two new sets of objects rather than a series (Ferry et al. 2015). In this case, there are contingent, salient, perceptual features that attract an infant’s attention and hamper the abstraction of the relationship. Similar results were found in studies involving acoustic stimuli, such as vowel and consonant sounds (Kovács and Endress 2014; Hochmann et al. 2018, 2011) and also with an anticipatory eye movement paradigm (Addyman and Mareschal 2010, esp. 2). In the latter case, pairs of shapes which were either the same or different moved together behind an inverted, T-shaped occluder; the shapes reappeared on one side if the shapes were the same and on the other side if they were different. If the infants, when presented with novel stimuli, correctly anticipated the side on which the shapes would reappear, this was taken as evidence that they had learned the underlying same–different relationship; they were in fact already able to complete this task at 8 months of age. All of these findings may be interpreted as evidence that human infants have a pre-linguistic, relational processing mechanism that allows them to compare examples and determine whether they are the same or different^1^. The acquisition of language then amplifies this ability, making it possible for individuals to deal with relationships which are beyond the superficial similarity of exemplars (e.g., Du et al. 2018; Gentner and Hoyos 2017; Hespos et al. 2020; Hochmann et al. 2017).

The abovementioned findings demonstrate that many non-human animal species and 3–7-month-old human infants can distinguish sameness from a lack of sameness, and this represents a solid starting point. However, a lack of sameness can take various forms. Two shapes, objects or movements that are not the same can be similar to each other, different to each other or opposite to each other. We have an intuitive understanding of what this means. In everyday life, this distinction is widely used and obvious (“These glasses are similar to the ones I have, but not the same”, or “This hotel room is completely different from the one I saw on the website when I booked it”; or “Be careful! You are driving in the opposite direction to where we need to go!”). While we all intuitively know that these three relationships are not the same, it is less obvious if we then try to understand the specific characteristics underlying the configurations which make us perceive them in three different ways. Clarifying this issue requires focused research.

### 1.2. Similar, Different or Opposite?

Since the origin of Psychophysics as a discipline, it has been acknowledged that there is a close link between similarity and perceptual mechanisms; since the origin, methods that required the participants to quantify the degree of similarity between two stimuli using rating scales were widely used. On the basis of similarity ratings, we can infer the distances between stimuli in a representational space and determine the most relevant dimensions relating to this space (e.g., Tversky 1977; Rosch and Mervis 1975; Nosofsky and Palmeri 1997; Smith and Medin 1981; Lin and Luck 2009).

Wertheimer (1923) acknowledged that similarity is one of the basic factors for perceptual organization: the parts of the visual field that are similar tend to be unified and segregated from the rest of the elements in the visual field. He included similarity in a list of factors, together with proximity, common fate, good continuation and closure, but later, discussions (e.g., Arnheim 1954, p. 54 ff; Palmer 1983, 1999, p. 258 ff; Vicario 1975) emphasized that similarity is in fact the only relationship needed to explain perceptual organization since it is implied in all other perceptual relationships: proximity implies a similar location, common fate involves similarities in motion and direction and so on.

In all of these studies, similarity enters the experimental design as an independent rather than a dependent variable. As noted by Palmer (1999, p. 276), Wertheimer postulated that the existence of this relationship between the elements in a scene would determine its visual organization, but he did not study similarity per se. A few years later, it was Goldmeier ([1936] 1972 translated by Rock in 1972) who, for the first time, systematically analyzed the characteristics of patterns perceived by observers as being similar. He asked participants to make similarity judgements for pairs or groups of figures. He discovered, for instance, that in order to predict which configurations would be considered more similar to the standard one, it is not enough to count the number of parts which are identical. Preserving the grouping of the parts of the stimulus is important in order for similarity to be perceived. He also found that proportional changes to all of the individual parts of a figure result in a more similar figure to the standard figure than disproportional changes; he also found that changes applied to singular, pregnant features (e.g., right angles; symmetry or orientation along the main spatial axis) impact the perception of similarity more than changes to a similar degree applied to non-singular features. In fact, a clockwise rotation of 5° applied to a vertical, straight line is perceived as a more abrupt change than the same rotation applied to a line which is already rotated 10° clockwise with respect to the vertical axis. In the 1970s, some of these findings aroused the interest of a number of noteworthy psychologists such as Rock (1973), who refocused on the role of orientation in the perception of similarity between shapes; and Palmer (1978), who refocused on the role of the number of identical elements versus the conservation of global aspects. Tversky (1977) extended this investigation not only to comparisons between perceptual stimuli, but also to comparisons between concepts. He then formalized the idea that similarity depends on a precise ratio between common and distinctive features. He also noted that the impact of distinctive features is not the same for judgments of diversity as compared to similarity, thus suggesting that similarity and difference are not simply inverse measures.

All of these studies focus on what naïve observers immediately recognize as being similar or different when they compare two or more stimuli. In this sense, the judgments were phenomenal; that is, they were based on the perception of a precise relationship relating to a given configuration. This is in agreement with the perspective we take in this paper. Later on, analyses of these relationships shifted away from naïve perception towards shape-recognition or pattern-recognition models, and the focus was no longer on the type of difference that a naïve observer perceives, but on the processes underlying the recognition of two patterns as being the same or different^2^. However, in the abovementioned studies, the interest was also oriented mainly towards similarity and only marginally towards diversity^3^, and any reference to opposition is totally absent. A legitimate question to ask is whether humans can recognize opposition between two visual or acoustic configurations and, more generally, between two phenomenal experiences just as they do for similarity and diversity.

This issue was somehow in the minds of the founders of Experimental Psychology, both in Wund’s perspective and in Meinong’s and Ehrenfels’s phenomenological perspective (as shown by Bianchi and Savardi 2008a, pp. 23–28). However, the topic then disappeared from experimental investigations in the field of Psychology until recent times, but in the meantime, it became an important topic within language studies. We cannot, however, be in too much of a hurry to conclude that this disciplinary shift is a sign that opposition—unlike sameness, similarity and difference—is purely a matter of language. In the present paper, we aim to support this statement first by revising some of the literature that suggests it is, in fact, not the case that opposition is a mere linguistic issue (Section 1.3); then, we present the results of two empirical studies (Section 2 and Section 3).

### 1.3. A Mainstream Approach to Opposites as a Semantic Relationship and Some Recent Moves towards a Perceptually-Based Perspective

From the 1970s onwards, the study of opposition has basically been developed from a linguistic and semantic point of view (Croft and Cruse 2004; Cruse 1986, 2000; Fellbaum 1995; Jones 2002; Murphy 2003). With the shift of the study of linguistics towards cognitive linguistics, significant changes occurred in the methods used to study this relationship. Rating tasks, elicitation tasks, response times, eye movements and so on have all been used, in addition to corpus-based data, to investigate the phenomenon (e.g., Jones et al. 2012; Paradis 2016; Paradis et al. 2015). New questions have arisen, and specific attempts have also been made to connect the meaning of antonyms to the perceptual experience of the properties that these antonyms describe (e.g., Bianchi et al. 2011; Bianchi et al. 2017a; van de Weijer et al. 2023). An interesting observation that recurrently appears in the literature on the subject is that opposites have a special status among other semantic relationships, and some authors have, in fact, referred to the “unique fascination” of antonyms (Cruse 1986, p. 197; Jones 2002, p. 1). It is the only semantic relationship “to receive direct lexical recognition in everyday language. In this sense it is more widespread and more primal than other relations holding between words” (Jones 2002, p. 8; for a similar observation see Cruse 2000, p. 167). It is intuitively and naturally understood and learnt, even though people cannot give an explicit definition of what makes two things opposite (Hermann et al. 1986; Kagan 1984; Miller and Fellbaum 1991; Murphy and Andrew 1993; Paradis et al. 2009).

One of the frequently mentioned characteristics of opposites is that they lie at the two ends of an underlying, common dimension. The easier the identification of this dimension, the quicker and more consistent the identification of the two words as opposites is (e.g., Hermann et al. 1986; Paradis et al. 2009). Whether these considerations (i.e., that there is evident contrast in an otherwise invariant overall structure) are purely semantic or whether they rather connect to certain characteristics that humans perceptually experience as being opposites is an interesting question to ask. These sorts of questions have inspired a number of studies in which the aim was to define the features of visual and acoustic stimuli that people recognize as opposites (see Bianchi and Savardi 2008a for an introduction to this perspective). What emerged from these studies is that when people look at, for example, their bodies or at another person’s body in a plane mirror, they typically associate opposition rather than similarity or diversity with the relationship they see. This was explored with various orientations of the mirror with respect to the body and also with varying postures of the person in front of it (Bianchi and Savardi 2008b). Similar outcomes were found when movements in front of the mirror and non-parallel to the mirror surface were concerned; or when simple everyday objects with an asymmetrical structure were placed along the axis orthogonal to the mirror (Bianchi et al. 2015). Opposition is so pervasive in people’s naïve experience of mirrors that it is also at the basis of some errors that they make when they are asked to predict the behavior of a reflection (Bianchi et al. 2015; Bianchi and Savardi 2014). Furthermore, it appears that adults generally associate opposition with the idea of symmetry. The naïve representation of a symmetrical configuration does not only contain sameness, but also the presence of a visible opposition between the parts forming the symmetrical pattern (Bianchi et al. 2017b). This is also an interesting aspect if we take into consideration the fact that in same–different tasks, pairs of symmetrical objects are sometimes used as exemplars of “the same”, as are stimuli showing two identical objects. Opposition was also associated with a specific kind of mirror transformation when acoustic stimuli formed of a series of notes were considered (Bianchi et al. 2017c). Finally, in simple motor tasks for which the participants were asked to perform the opposite gesture with regard to a target model or to rate the extent to which the gestures performed by two individuals were opposites, the responses were very consistent (Bianchi et al. 2014). Despite the fact that it had not been specified how “doing the opposite” was supposed to be interpreted, the participants did not in fact vary many of the features of the gestures, but rather altered aspects concerning its overall orientation. Furthermore, it was found that doing the opposite of a gesture did not require more time than making the same gesture. This is interesting since it supports the idea that opposition is intuitive.

A common result which emerged from these studies regards the fact that two configurations that were perceived as opposites were characterized by a high level of invariance and that the critical difference concerned the spatial characteristics of the figure, mostly in terms of its allocentric structure in space. This also seems to hold for other pilot studies conducted with both adults and 7- to 8-year-old children who were asked to draw the opposite of a simple bidimensional figures or to rank series of pairs of figures, from the most to the least opposite (these pilots are reported in Bianchi and Savardi 2008a, pp. 95–100, 115–30). Based on these premises, we designed the two studies presented in this paper.

## 2. Study 1

In Section 1.2, we mention some prototype studies dealing with relationships as perceptual data (Goldmeier [1936] 1972; Medin et al. 1990; Palmer 1978; Rock 1973; Tversky 1977). From a methodological point of view, these studies all used recognition tasks and simple visual configurations in order to identify the rules underlying the perception of similarity between two configurations. Participants were shown pairs of stimuli or a standard figure and a series of comparison figures and were asked to judge their similarity. In the present study, we used essentially the same methods to ascertain the characteristics of the pair of figures which were recognized and described by participants as being opposites and to assess how they differ from the pairs recognized as being similar or different.

The participants were shown a series of simple figures which consisted of a standard shape on the left and paired with a comparison shape on the right. In three separate sessions, they were asked to rate the degree to which they perceived the comparison shape to be either similar (S), different (D) or opposite (O) with respect to the standard shape (see the methods section for details of the procedure).

The study had two main goals. The first was to investigate whether significant differences emerged between the S, D and O, which would mean that the participants were basing their responses on different criteria. Our main interest focused on opposition. We hypothesized that if opposition is a specific relationship, which is not reducible and does not overlap with either of the other two relationships, this would emerge both in a synthetic way when we explored the correlations between the whole series of S, D and O ratings and also analytically when we assessed the S, D and O ratings given to each individual pair of figures.

The second goal was to study the relative effects of the transformations applied to the standard figures on the S, D or O ratings. In particular, we aimed to understand whether there are specific transformations among those considered in the study which are associated with significant changes in the O ratings; and any which do not (and likewise for S and D).

### 2.1. Method

#### 2.1.1. Participants

A total of 187 participants took part in the study (120 females, 64%, 67 males; mean age: 24.636; sd: 7.834). They were recruited at the University of Verona at the beginning of a Psychology course on topics unrelated to the study. They volunteered to participate in the study.

#### 2.1.2. Materials

**Figure 1:**
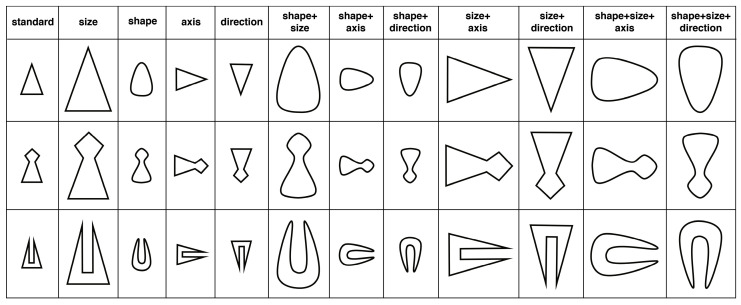
The standard figures used in the study and the transformations displayed in the comparison figures.

We used three two-dimensional figures as the standard shapes (see first column in Figure 1). They were characterized by a comparable spatial structure: they were all angular rather than rounded; the main (i.e., the longest) axis was vertical; and they were oriented upwards. For each figure, there were twelve comparison figures, eleven of which were obtained by transforming either one, two or three of the features of the standard figure. From a phenomenal point of view, the transformations consisted of a change in the shape of the contour (i.e., from angular to rounded), the size of the figure (i.e., from small to large), the axis of orientation (i.e., from vertical to horizontal) and the direction of orientation (i.e., from pointing upwards to pointing downwards)^4^. These four basic transformations were applied either singly or combined with other transformations. The transformations regarding the axis or the direction were the only two that were never applied together since this is not possible, but they were presented in association with all of the other transformations. An example of each transformation is shown in Figure 1.

The set of stimuli presented to participants consisted of pairs of figures formed of a standard stimulus (always to the left and indicated by the letter A underneath it) and a comparison figure (always to the right and indicated by the letter B underneath it). There were 12 pairs in total for each standard figure. There were eleven comparisons with transformations and a pair for which the comparison stimulus had not undergone any transformation with respect to the standard figure (we will refer to this as the identity pair).

To conduct the experiments, we used a customization of LimeSurvey (Limesurvey GmbH 2023), an open-source online survey application that allows users to create, publish and collect data. LimeSurvey is built using the PHP programming language and a MySQL or PostgreSQL database for data storage. It follows a client–server style architecture, and it is accessed by users by means of a web browser. The application was responsive; that is, it was possible to adjust its layout, content and the functions based on the device or screen being used to access it.

#### 2.1.3. Procedure

The experiment was carried out online at the beginning of a class on a topic which was unrelated to the study. The participants accessed it individually using their own devices (i.e., PC, tablet or smartphone).

The participants were given information about the task and were asked to complete an informed consent form on the first page. Some personal data (i.e., gender and age) were then requested on the second page, and the experiment started on the third page. Instructions regarding the relationship to focus on during the experiment (i.e., S, D or O) were shown in capital letters at the center of the screen. The participants rated the relationship between the stimuli separately (i.e., either S, D or O). The order of the three relationships was randomized among the participants.

The pairs of figures to be rated appeared at the top of the screen in the center with a question underneath: “To what extent does figure B appear to be similar [or different or opposite] to figure A? (0 = not at all; 10 = completely) Move the cursor to respond”.

No time limits were set. After the participants had given their rating, they pressed a button (“Next”) and a new pair of stimuli appeared, followed by the same question and the min–max cursor. For each of the three relationships considered, that is, similar (S), different (D) and opposite (O), the participants were shown 36 stimuli in total (i.e., 3 standard figures x 12 comparison stimuli for each standard figure). They were randomized between participants. The participants completed their responses for each relationship before moving on to the next relationship. The experiment ended when the participants had given their ratings for each of the three relationships (S, D and O). The time taken was approximately 25 min.

The responses were recorded by means of MySQL and exported to an R database. The study was conducted in accordance with the Declaration of Helsinki, and the protocol was approved by the Ethics Committee of the University of Verona (Prot. n. 161865, 17 April 2023).

#### 2.1.4. Data Analyses

All statistical analyses and mathematical calculations were performed using the R statistical software, version 4.3.0 (R Core Team 2023). In particular, the correlations were calculated by means of Pearson’s r index (psych R-package; Revelle 2023). The Linear Mixed-effects Models (LMMs) were performed using the R-packages lme4 (Bates et al. 2015), stats (which is part of the standard R software) and emmeans (Lenth 2023). The cluster analyses were conducted using the R-packages stats and factoextra (Kassambara and Mundt 2020). The power analysis used the functions of the R-packages pwr, WebPower (Zhang and Mai 2023) and performance (Lüdecke et al. 2021). The plots for the analyses were created using the ggplot2 R-package (Wickham 2016).

### 2.2. Results

**(a) The correlations between the ratings relating to S, D and O.** An analysis of the correlations between the ratings relating to S, D and O (made by averaging over participants and over standard stimuli) provided a first overall picture of the mutual relationship between them. There was a very strong, almost perfect, negative correlation between S and D (r = −0.975, *p* < 0.001). There was a negative correlation between O and S (r = −0.614, *p* = 0.044), but this was not as strong as that between S and D. The relationship between O and D showed only a trend towards a positive correlation (r = 0.572, *p* = 0.066).

These results indicate that the three relationships are not independent. However, at the same time, they also indicate that the ratings relating to O are in a specific relationship with S and with D, which is not the same as that which can be observed between S and D.

**(b) An analytical picture of the 11 transformations: how often did the O ratings significantly differ from the S and D ratings?** In order to carry out a more detailed analysis of the difference between the three types of rating, it was necessary to consider the responses given with regard to the eleven transformations separately. A type III analysis of variance with Satterthwaite’s method for a Linear Mixed-effects Model (LMM; Gaussian family; identity function) was conducted on the ratings given by the participants.

Type of Relationship (S, D, O) and Transformation (on 11 levels) were the two fixed effects studied in the LMM. Since we were interested in the interaction between these two variables, the choice of the model was theory-driven rather than the result of a data-driven comparison between models. Standard figure and participants were the random effects. For the purposes of the hypotheses tested in our study, the standard figures used were simply exemplars of a general category (i.e., elongated angular figures pointing upwards) and they were interchangeable with any other figure of the same type. The participants entered the model as a random effect since we had a repeated measure design. We used a crossed design (not a nested one) since the levels of our fixed effects (Transformations and Type of Relationships) were always the same for all of the standard figures and for all of the participants.

**Figure 2:**
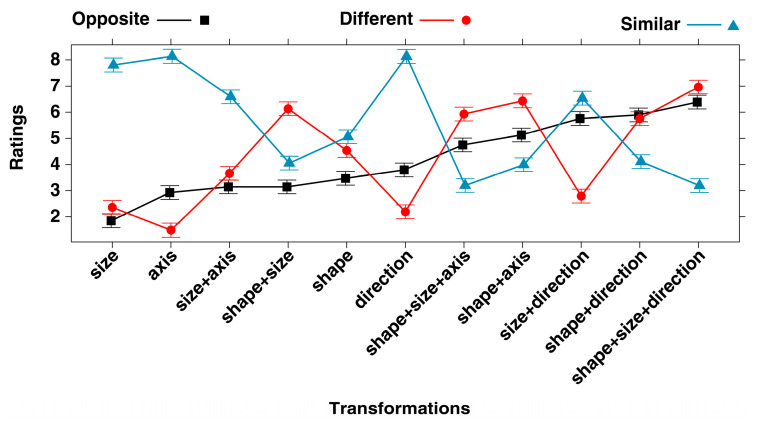
Effect plot on ratings of the interaction between Type of Relationship (similar, different, opposite) and Transformation (on 11 levels). On the x-axis, the order of the 11 transformations used in the study is based on the O ratings, from the least to the most opposite. For a visual example of the transformations, see Figure 1. Error bars represent the 95% confidence interval.

The interaction between Type of Relationship and Transformation turned out to be significant (F(20, 18,087) = 425.993, *p* < 0.001; conditional R-squared = 0.399; power (alpha = 0.05) = 1.000; Standard figures: ICC = 0.002; Participants: ICC = 0.096). The effect plot of the interaction is shown in Figure 2. Along the x-axis, the eleven transformations are ordered according to the O ratings, from the least to the most opposite. As one can see in the figure, the O ratings in most cases do not overlap with the S and D ratings. Bonferroni post hoc tests (Table 1) confirmed that the O ratings were significantly different from both the S and D ratings for 8 out of the 11 transformations. For the remaining three transformations, they differed from S but not from D.

**Figure 3:**
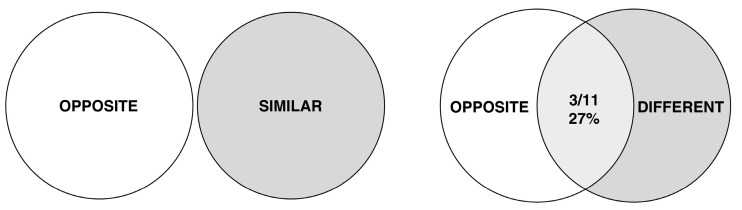
Euler–Venn representation of the relationship between the set of O ratings and the sets of S and D ratings. Each set includes 11 members corresponding to the 11 transformations presented in the study. The representation displays the picture which emerged from the post hoc tests presented in Table 1.

Figure 3 shows a visual summary of the results. The intersection between the S and O ratings is null, while the D and O ratings overlap in three cases.

**(c) Which transformations had a greater effect on the O ratings? And which had a greater effect on the S and D ratings?** The effect of each of the 11 transformations on the three types of rating (S, D, O) was estimated in terms of the difference between the rating given to the transformation in question and the rating given to the identity pair: that is, the pair with a comparison stimulus which is identical to the standard stimulus^5^. Cohen’s d coefficient was used to define the effect size (considering d in terms of absolute value: 0 < d < 0.2 is considered a very small effect; 0.2 < d < 0.3 is a small effect; 0.3 < d < 0.5 is a medium effect; d > 0.5 is a large effect). In Table 2, the size of the effect is indicated by the numbers 1 (big), 2 (medium) and 3 (small) in parentheses. The z-ratio in the last three columns refer to the post hoc test of the comparisons between the rating relating to the transformation in question and that of the identity pair.

As shown in Table 2, all of the transformations had a significant effect (either medium or large) on S in the sense that they reduced the degree of similarity attributed to the identity pair to varying extents (hence the negative values for Cohen’s d in the 6th col.). Likewise, all of the transformations had a significant, but in this case positive, effect on the D ratings (either medium or large); namely, they increased the D rating given to the identity pair to varying extents. Three of the transformations had no significant effect on the O ratings. That is to say, the single transformation relating to the axis of orientation (from vertical to horizontal), the same transformation combined with a change in size (from small to large), and the transformation in size in combination with that of the contour (from angular to rounded) did not lead to greater or smaller ratings of opposition with respect to the identity pair. In all other cases, a significant effect emerged. The effect size was in general smaller than that for the S and D ratings (almost all cells in Table 2 are marked with (3)). Only the combined transformation in direction and size (size+direction) had a medium (rather than small) effect on the O ratings. Interestingly, the same transformation only had a small effect on the D ratings. As can be seen in Figure 2, the three transformations that received an O rating which was significantly higher than the D rating are those involving a single transformation of the axis of orientation or of the direction of orientation. In the latter case, this referred to both single transformations or those in combination with an enlargement in size (size+direction), but always without any transformation in the contour (i.e., from angular to rounded, shape+). When a transformation of the contour was involved, this tended to receive a higher D than O rating (see, in Figure 2, the shape+size, shape, shape+size+axis, shape+axis, shape+size+direction transformations). Only when a change in contour was associated with a change in direction (shape+direction) was the extent to which they were perceived to be D and O similar.

An analysis of the results shown in Figure 2 also revealed that a transformation in size did not seem to have more of a specific effect in terms of O than D or vice versa. Its effect was somewhat secondary to the presence of a combined transformation involving direction (size+direction), in which case the participants perceived more O than D; or a combined transformation involving the contour, in which case, conversely, the participants perceived D rather than O (see shape+size, shape+size+axis, shape+size+direction in Figure 2).

**Figure 4:**
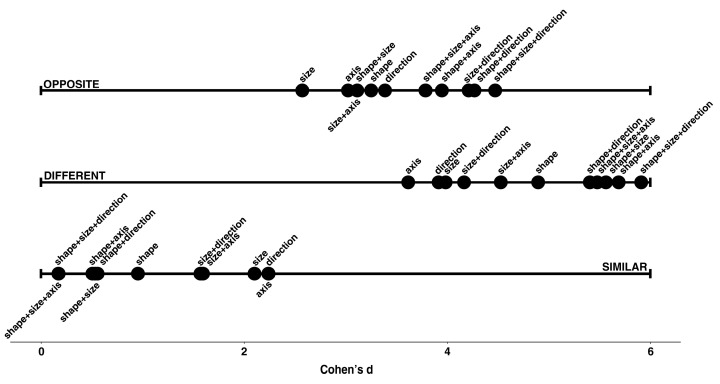
Unidimensional scalings relating to the transformations based on Cohen’s d effect size, for each of the three relationships.

These observations were confirmed when we looked at the unidimensional scalings relating to the 11 transformations based on the effect size, considered separately for each of the three types of relationship (Figure 4). The transformations with the greatest effect in terms of D all included a transformation in shape (see the six transformations to the extreme right of the D scale). These were also the transformations that, on the contrary, had the greatest negative effect in terms of the perception of Similarity. Conversely, the three transformations that had the greatest effect in terms of O were all characterized by a change in direction (i.e., an upside-down transformation).

**(d) Additive versus non-additive effects on the S, D and O ratings of combining transformations.** The stimuli were created initially with single transformations in contour, size, axis or direction, and then, with a combination of these. However, judgments involving the participant’s perception, such as those in the present study, are most likely influenced by the overall global impression. An interesting aspect to investigate is therefore whether the three types of relationship (S, D and O) also differ in terms of the extent to which they conform to an “additive” logic. This means investigating, for example, whether a D rating given to a stimulus that combines a transformation in size and contour (shape+size) is significantly different from the rating that would be obtained by adding together the ratings given to the two transformations individually (i.e., shape [+] contour).

We studied this issue by analyzing the difference between the expected ratings (i.e., expected according to the hypothesis that the combined rating is the combination of the individual ratings relating to the transformations) and the actual ratings. For the seven stimuli in the study which represented a combination of two or three transformations and for each participant, we calculated the expected ratings starting from the ratings that each participant gave the individual transformations (relating to size, shape, axis and direction). We did this for each of the three relationships (S, D, O).

**Figure 5:**
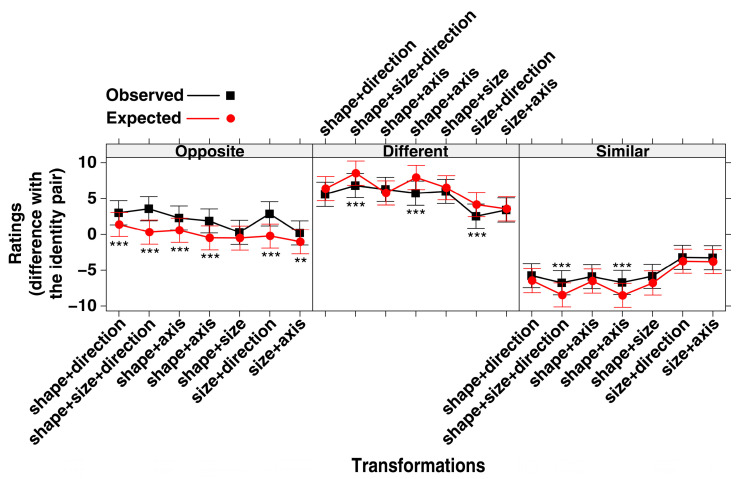
Effect plot of the interaction between Type of Relationship, Transformation and Expected/Observed Rating. Error Bars represent the 95% confidence interval. The asterisks indicate the stimuli for which a significant difference emerged. ** *p* < .01; *** *p* < .001.

A type III analysis of variance with Satterthwaite’s method for a Linear Mixed-effects Model (LMM, Gaussian family; identity function) was performed, with Type of Relationship, Transformation and Expected/Observed Rating as fixed effects; and Participants and type of Standard figure as random effects. In this case too, the choice of the model was theory-driven since we were interested in the interaction between the three variables. The interaction between Type of Relationship, Transformation and Expected/Observed Rating turned out to be significant (F(12, 20,442) = 10.654, *p* < 0.001; conditional R-squared = 0.554; power (alpha = 0.05) = 1.000; Standard figures: ICC = 0.123; Participants: ICC = 0.051). As Figure 5 shows and Bonferroni post hoc tests confirmed, the three relationships differ.

For O judgments, the Observed and Expected Ratings were significantly different for six out of the seven stimuli considered (therefore, responses do not comply to an additive logic). In all six cases, the value observed was greater than the theoretical expected value (obs. > exp. = 7 cases).

For D judgments, the Observed and Expected Ratings did not differ for four out of the seven stimuli considered. In the case of the three stimuli for which a difference emerged, the observed value was smaller than the expected value (obs. < exp. = 3 cases).

For S judgments, the Observed and Expected Ratings were the same for five out of the seven stimuli. In the case of the two stimuli for which a difference emerged, the observed value was greater than the expected value (obs. > exp. = 2 cases).

## 3. Study 2

Study 2 was designed to verify the robustness of the main results obtained in Study 1. The results of the first study indicated that the behavior of O was different with respect to S and D, in contrast to the strong negative correlation between S and D; and the effect of some transformations specifically in relation to D, in contrast with others which were more evidently associated with O. In Study 1, the participants were required to provide a quantitative response (i.e., a rating) for the three relationships. In Study 2, we used a pair comparison task which required participants simply to choose, out of two matched stimuli, which appeared to them to be more similar (or different or opposite) as compared to the standard figure.

There are several aspects which make this type of task advantageous (David 1988; Pinger et al. 2016; Luce and Tukey 1964; Thurstone 1927).

– Simplicity: it is a relatively straightforward method that is easy to understand. The participants are shown two options and asked to make a judgment about which option is better in some way (e.g., which appears to be more “similar” to the standard figure). In this way, it is accessible to a wide range of individuals and minimizes the potential for confusion or bias.
– Elimination of absolute scales: Unlike rating scales or Likert-type scales that require participants to assign a numerical value or rate each option individually, paired comparison tasks focus on relative judgments. Participants only need to compare two options at a time, which simplifies the decision-making process and reduces cognitive load. This approach can help overcome potential biases associated with absolute scales and facilitate more accurate and meaningful comparisons.
– Improved discrimination: Paired comparisons can enhance the sensitivity and discrimination of judgments. By presenting options in pairs, participants are forced to make direct comparisons and identify the relative differences between the options. This can lead to more precise rankings especially when comparing complex or nuanced stimuli.
– Reduced response biases: Traditional rating scales can be subject to various response biases, such as central tendency bias (tendency to select neutral or middle options) or acquiescence bias (tendency to agree with statements). The paired comparison task can minimize these biases by asking the participant to focus on relative judgments.
– Robustness: The paired comparison task is known for its robustness and its adaptability to a wide range of stimuli and contexts.
– Quantifiable results: Paired comparison tasks provide data that can be easily quantified and analyzed. The relative rankings obtained from participants’ judgments can be statistically analyzed to determine the overall preferences or rankings of the options being compared.

We did not have the impression that the participants in Study 1 found the task difficult; therefore, we did not expect a great deal of difference in Study 2. However, we considered that if the results were consistent across different methodologies, this would strengthen the robustness of the conclusions we reached.

### 3.1. Method

#### 3.1.1. Participants

A total of 130 participants took part in the study (68 females, 52%, 62 males; mean age: 25.145; sd: 7.211). They were recruited at the University of Verona at the beginning of a Psychology course on topics unrelated to the study. They volunteered to participate in the study. None of the participants in Study 2 had taken part in Study 1.

#### 3.1.2. Materials

We used a subset of the stimuli used in Study 1: in particular, the first standard figure represented in Figure 1 and its corresponding 11 transformations. The size of the visual stimuli was the same as that used in Study 1. The standard figure was always shown in the center at the top of the screen, and the two comparison figures were shown below and slightly to the left and right of the standard figure at an equal distance from it. For each of the relationships being considered (i.e., S, D and O), the participants were shown 55 paired comparisons. The stimuli were the same size as those used in Study 1.

#### 3.1.3. Procedure

The experiment was administered online. Participants accessed it individually using their own devices.

They were first given information about the task and asked to agree to an informed consent request for participation (on the first page of the online form). Personal data (gender and age) were collected on the next page and the experiment started on the third page.

The instructions appeared at the top of the screen. For S, for example, the participants were told to “Choose which of the two figures below appears to be more similar to the standard figure above and click on it”. The same instruction was adapted for D and O. After they had given their response, the next stimulus appeared. Fifty-five stimuli were shown one at a time under the instruction. They were randomized between participants for each target relationship (S, D and O). The target relationships were also randomized between participants.

The study was conducted in accordance with the Declaration of Helsinki, and the protocol was approved by the Ethics Committee of the University of Verona (Prot. n. 161865, 17 April 2023).

#### 3.1.4. Data Analyses

The paired comparison method used in Study 2 produced an interval z-score scale based on the participants’ responses to the stimuli. Each stimulus was assigned a z-score based on the number of times it was preferred to the other comparison stimulus in the pair. The stimulus with the highest number of preferences was ranked first, followed by the second and so on (Thurstone 1927).

All statistical analyses and mathematical calculations performed on the z-score scales were conducted using the R statistical software, version 4.3.0. In particular, the paired comparison method was conducted using the eba R-package (Wickelmaier and Schmid 2004). The correlations were calculated using Pearson’s r index (psych R-package). The cluster analyses (method K-means, Hastie et al. 2009; MacQueen 1967) were conducted using the R-package stats and factoextra. A combination of the elbow method with the average silhouette method and the gap statistic method were used to determine the optimal number of clusters (Kaufman and Rousseeuw 1990; Still and Bialek 2004; Tibshirani et al. 2001). The plots for the analyses were created using the ggplot2 R-package.

### 3.2. Results

The scalings for S, D and O all had a valid goodness of fit statistic (−2 log likelihood ratio, Critchlow and Fligner 1991). Precisely: goodness of fit (45) = 45.338, *p* = 0.458 for S; goodness of fit (45) = 48.771, *p* = 0.324 for D and goodness of fit (45) = 53.234, *p* = 0.209 for O.

This outcome allowed us to then proceed with an analysis of the results according to our research hypotheses.

**(a) Correlations between the S, D, O scalings.** As in Study 1, we analyzed the correlation between the scalings relating to S, D and O (performed by averaging over participants and over standard stimuli) in order to study the mutual relationship between them. S and D turned out to have a very strong, almost perfect, negative correlation (r = −0.982, *p* < 0.001). This was the only significant correlation which emerged from the results. No significant correlation was found between O and S or between O and D. In both cases, the direction of r was consistent with Study 1 (that is, a negative correlation between O and S and a positive correlation between O and D) but it did not reach significance (O and S: r = −0.479, *p* = 0.163; O and D: r = 0.452, *p* = 0.162).

These results confirm the strong relationship between S and D which was found in Study 1, and the relative independence of O from S and D.

**(b) The correlations between the average ratings (Study 1) and scalar values (Study 2) of the 11 transformations, assessed individually for S, D and O.** For each of the 11 transformations relating to each of the three relationships considered, the r-Pearson correlation coefficient r was calculated with reference to the mean values obtained in the rating task (Study 1) and the scalar values obtained in the paired comparison task (Study 2). The correlation (performed by averaging over participants and over standard stimuli) turned out to be highly significant for all three relationships: S (r = 0.836; *p* < 0.001), D (r = 0.882; *p* < 0.001) and O (r = 0.888; *p* < 0.001).

**Figure 6:**
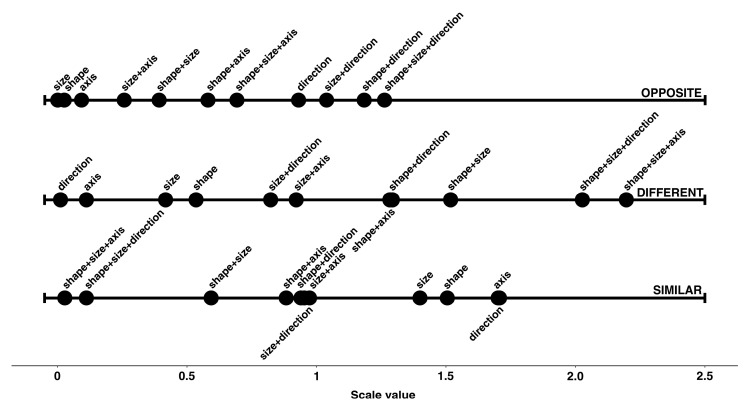
Unidimensional scalings for the three relationships. The scalar values are expressed in z-scores and are the result of applying the paired comparison method.

**(c) The scalings relating to the eleven transformations in Study 2, analyzed separately for each of the three relationships.** The scalar values expressed in z-scores (obtained as the output from the application of the paired comparison method) were represented on three different unidimensional scales: one for each relationship (Figure 6). The picture which emerged was highly consistent with the results found in Study 1 (Figure 4). The transformations associated with a stronger perception of O all show a transformation involving the direction of orientation (i.e., upwards–downwards). A stronger perception of D was associated with the five transformations that involved the shape transformation (combined with another transformation). These are the same transformations which were associated with a weaker perception of S, while a stronger perception of S was associated with a single transformation in only direction, size or shape.

**(d) Joint analysis.** The final analysis we carried out was a three cluster analysis (method K-means) with the aim of exploring how the transformations clustered together based on a combination of the results from Study 1 and Study 2.

**Figure 7:**
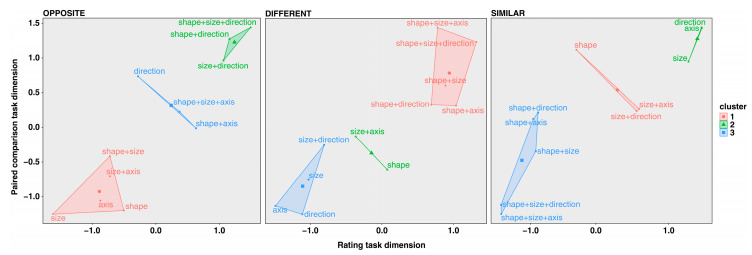
Cluster analyses (K-means method) on the results of Studies 1 and 2 (jointly) for each of the three relationships (S, D and O).

By combining the elbow method with the average silhouette method and the gap statistic method, we found three clusters for each relationship (S, D and O). With reference to the O relationship, the results accounted for 87.5% of the total variability in the data. With reference to D, the clustering accounted for a proportion equal to 92.2% of the total variability in the data. With regards to S, the clustering accounted for a proportion equal to 91.8% of the total variability in the data. The results are shown in Figure 7.

In the results relating to O, there was one cluster which grouped the three transformations involving an inversion of direction (from up to down) in combination with a transformation in size and shape, either singly or combined. These transformations were associated with the maximum perception of opposition. A median cluster groups the single transformations involving direction and two transformations involving a variation in the axis in association with other features (shape and shape+size). In the third cluster (minimum opposition), there are single transformations relating to shape, size, and axis of orientation; and the double transformations consisting of shape+size and size+axis.

With regards to D, one cluster groups all of the transformations involving a variation in the shape of the contour in combination with other variations. These were the transformations which were perceived as being maximally different. The transformations in the axis of orientation, the size and the direction (carried out singly or in combination with size) were associated with the perception of a lower degree of diversity. The cluster including transformations in shape alone and combined transformations in size and axis of orientation was perceived as showing a slightly higher degree of diversity as compared to the latter cluster, but with the result still a long way from that relating to the former cluster.

A picture which is basically inverted emerges from the cluster analysis which related to S, at least as far as the cluster which groups the less similar stimuli is concerned. It groups the same stimuli as those belonging to the cluster with the most diverse (see the central plot referring to D). Three transformations (that is, the single transformations in size, axis and direction) form a cluster with the stimuli which were considered to be the most similar. The transformations in shape alone and in the axis or the direction in association with a transformation in size form an intermediate cluster.

The position of the transformation in direction alone in the three plots in Figure 6 is interesting: an upside-down inversion leaves most of the overall identity between the two figures invariant, and this is why it was judged as having a very high level of S and an extremely low level of D. However, at the same time, these specific variations were associated with a clear perception of O.

## 4. Final Discussion

The purpose of this paper was to shine a spotlight on the perceptual foundations of the relationships which are the basic building blocks of human cognition, namely sameness, similarity, difference and opposition. The goal was relatively straightforward for the first three relationships. It was simply necessary to (a) remember that a basic construct on which Psychophysics was and is founded concerns the discrimination of the sameness/difference and similarity/difference relating to sensations and (b) acknowledge the consolidated streams of research on the foundations of same/different discrimination and abstract generalization in pre-linguistic infants and some non-human animal species (Section 1.1 and Section 1.2 of the Introduction to this paper). For the fourth relationship, that is, opposition, the goal was achieved by referring to the findings of a number of previous studies which demonstrated that certain visual and acoustic configurations are recognized by observers as being opposites rather than similar or different (Section 1.3). We also added new empirical data by means of two studies which focused on comparisons between simple bidimensional figures (Section 2 and Section 3).

There are, to date, still very few studies on the topic of opposition. Investigations of sameness, similarity and difference have proceeded apace in the field of Psychology, but we are still far from having investigated opposition to the same extent and depth. The presence of a rich reservoir of research usually compensates for the fact that each individual study necessarily involves a limited set of stimuli. This limitation also holds for the two studies presented in this paper, as we will discuss further on. Moreover, our two studies are not backed up by a great deal of previous research. However, having said that, there do seem to be three recurring outcomes relating to this paper which are in agreement with some earlier studies on opposition (Bianchi and Savardi 2008b; Bianchi et al. 2014, 2015, 2017b, 2017c). We will first briefly present these and then discuss the limitations of these conclusions which are due to the limitations relating to the studies.

Firstly, taken as a whole, the judgments of opposition do not overlap with those of similarity and diversity, and this can be considered to be an indication that this relationship is characterized by its own specificity. Secondly, the perception of opposition is specifically linked to the presence of two objects or parts of objects which, from an allocentric perspective, point in opposite directions. Lastly, an alteration to the shape of an object is not always compatible with the perception of opposition; it is more likely to be associated with diversity. Alterations in shape are only associated with opposition when there is also an inversion of direction (the latter seems to elicit the perception of opposition despite the change in shape).

The robustness and generalizability of these conclusions are subject to a series of limitations, some of which are already evident in the results of our studies. These relate in particular to the different methods used in Studies 1 and 2. The significant correlations which emerged between Studies 1 and 2 with regard to the judgments of O, S and D suggest that the results are overall generally robust and generalized across task. However, the results of the two studies were not totally in agreement, and there were, in fact, some differences. For instance, the single transformation concerning direction ranks higher, as compared to the other transformations, in the scaling pertaining to Study 2 (Figure 6) than in that pertaining to Study 1 (Figure 4). Furthermore, in Study 2, no significant correlation was found between the judgments relating to O and D and to O and S (while S and D were very highly correlated). In Study 1 however, the O ratings were always significantly different and negatively correlated to the S ratings, while the ratings relating to O and D did not turn out to be significantly distinct for 3 out of the 11 transformations considered. This was despite the fact that no significant overall correlation emerged between the D and O ratings, in agreement with the results from Study 2. The three transformations that did not receive significantly different D and O ratings were size and size+axis (both characterized by low O and D ratings) and the transformation involving shape+direction (which was characterized by high O and D ratings). In future research, it would be interesting to identify other cases in which D and O behave indistinctly and ascertain whether there are cases in which the judgments relating to S and O overlap. These last considerations lead us to another limitation concerning the specific set of stimuli used in these studies.

Since our main intention was to test the distinctiveness of the S, O and D judgments and the robustness of the results when different methods were used (i.e., rating versus pair comparison), we kept the experimental design simple with respect to other variables. For instance, we decided to test only figures pointing in a clear direction with respect to an axis of elongation. We did not include squares or rectangles, that is, figures lacking a main elongation axis or structural directionality. Likewise, the four basic transformations used in the study (i.e., relating to contour, size, axis and direction) were only applied in one direction, namely, from angular to rounded, from small to large, from vertical to horizontal and from upright to upside down, and not vice versa. Furthermore, we did not consider transformations in color or texture of the surface of the figures. We know from some of the pilot studies carried out by Bianchi and Savardi (2008a) that these characteristics are all potentially relevant. For instance, it was found that a transformation in size from small to large does not have the same effect (in terms of making the two figures look opposite) as the same transformation when it is applied in the inverse direction, that is, from large to small and so on. A similar anisotropy emerged in the case of a transformation from straight to bent and vice versa. It was also found that the axis of orientation assumes a more prominent role in perceptual judgments of opposition when the figures do not point in a clear direction (e.g., rectangles and squares).

All of these variables are worth considering in more detail in future studies which might want to concentrate on the difference in impact associated with various types of transformation on various different kinds of figures (e.g., with and without a specific direction). It would also be worth exploring the symmetry or asymmetry of the effects of these transformations when applied in both directions (i.e., going from small to large, and from large to small and so on). However, if there is a much larger set of stimuli, one might then be forced to consider only single transformations and not combinations of transformations in order to control the complexity of the experimental design and the length of the task. Alternatively, one might prefer to study within subjects the effects of both single and combined transformations, but for only one standard figure, for each type of transformation (S, D and O). For the main goals of our paper, we considered that it would be more relevant to test the three types of relationships within participants and to test the consistency of the judgments associated with the 11 transformations using different standard stimuli while limiting the transformations to only one direction and focusing exclusively on figures with a clearly defined vertical direction.

Furthermore, we are aware that another limitation of the study is that, according to conventional practices, we have looked into the data measuring statistical differences, but this is not the only approach possible (see Hanel et al. 2019). When comparing two or more groups of data, researchers often make the assumption that a lack of significant difference indicates a high degree of similarity, while a statistically significant result suggests a low level of similarity. We are, however, aware that while a null difference may potentially confirm (or at least not disprove) a high degree of similarity, a significant and/or substantial difference does not necessarily imply a lower degree of similarity. Research on topics such as those presented in this paper might benefit from considering both approaches to data analysis.

Before concluding, we would like to mention some implications of our results in relation to the main research domains discussed in the introduction. In particular, the finding that opposition is a specific relationship which is completely distinct from similarity and difference might prompt researchers to start reasoning triadically (i.e., in terms of similarity, diversity and opposition) rather than simply dyadically (i.e., only in terms of sameness vs. difference, or similarity vs. diversity).

One first consideration concerns the impact of the same/different distinction in infants’ abstract categorization. Analogical comparison is acknowledged as a major driver for the development of new abstractions in categorization. In their review paper, Gentner and Hoyos (2017) discuss the role of alignment in language acquisition processes and show that more exemplars are not always better for learning. According to them, the ease of detecting the relationship between the exemplars is the critical factor in terms of developing the ability to make correct generalizations derived from the stimuli presented for training purposes. Similarity has an important facilitating effect on this (Gentner and Hoyos 2017). Even for adults, relational mapping and transfer is facilitated by a high degree of overall similarity between matched situations/stimuli and is, conversely, hampered when the two situations/stimuli appear to be different (Gentner et al. 1993; Holyoak and Koh 1987; Ross 1987; Trench and Minervino 2015). This is even more true for children. Early on in the learning process (that is, from the first year to 9 years of age), an elevated degree of similarity is important not only for a match to be readily noted, but also for the ease of aligning the individual elements so that a comparison can be successfully made (Brown and Kane 1988; Chen et al. 1997; Gentner et al. 1993; Gentner and Kurtz 2006; Gentner and Toupin 1986). Similarity and diversity, respectively, therefore encourage or hamper new comparative processes.

But what about opposition? Does it facilitate or hamper comparison? This would be an interesting hypothesis to test, and it could be carried out by simply manipulating the kind of stimuli used in a habituation/dishabituation experimental paradigm and in the analogical transfer paradigm. Rather the foreseeing merely a “same” vs. a generic “not-the-same” condition, the experimental design would operationalize the latter in two distinct ways (i.e., opposite vs. different). It would thus be possible to observe whether and in what way infants’ performance changes when a “not-the-same” stimulus consists of something that humans tend to perceive as “opposite” rather than “different”. If the former type of non-sameness turns out to be easier to distinguish and abstract at this pre-linguistic level, then this would potentially pave the way to finding a new explanation for why antonyms are so primal in human language acquisition. When two linguistic labels apply to situations or experiences that, because of their perceptual structure, are easy to align and match and the contrasting aspect is easily noticed, then the abstract relationship that the two distinct words indicate should be easier to pick up and learn. For example, a small square and a colorful pencil are so clearly completely different that elements of mismatch between them are very difficult to identify and transfer. The same does not hold for a comparison between, say, a white square and a black square, or a triangle pointing up and a triangle pointing down. In other words, antonyms might have a special status as a semantic relationship because they label a type of non-sameness that is unambiguous. It is easy to notice in the training phase and, therefore, also easy to transfer elsewhere (as predicted by Gentner and Hoyos 2017, p. 687).

Furthermore, operationalizing non-sameness in terms of perceived diversity versus perceived opposition or perceived similarity might suggest a new way of interpreting specific findings concerning the performance of pre-linguistic infants and non-human animals related either to the training phase or the following phase involving the generalization of the relationship which has been learned to novel stimuli. For instance, some studies have found an increased ability of infants to more correctly generalize a different relationship compared to a same relationship (see Addyman and Mareschal 2010 for a review). As both Young and Wasserman (2002) and Smith et al. (2008) make clear, there is a great deal of asymmetry between the two cases. Infants might be responding to lower-level aspects of the stimuli (such as chromatic contrast, symmetry or variations in shape, etc.) which are considerably more present in stimuli which are different than in those which are the same. Because of this complexity, stimuli which are different may engage infants more, prompting them to learn more about this relationship than the relationship between things which are the same (Young and Wasserman 2002). A careful analysis of the features of stimuli which are different might reveal the conditions in which this asymmetry emerges, thereby clarifying the aforementioned interpretation. It may be that the asymmetry which emerged in certain studies but not in others is due to the type of stimuli representing non-sameness. In effect, we might ask if they were really similar or different or opposite.

Likewise, operationalizing non-sameness in terms of similarity, diversity or opposition might help us to rethink the explanation for the differences in the time needed by infants in the training phase to learn “same” and “different” relationships (e.g., Harding and Cousineau 2022; Goulet and Cousineau 2020). Is it more time consuming to learn relationships which are similar, different or opposite? The same questions also apply to studies with non-human animals. Configurations which are different are not the same as those which are opposite. What is the impact of the three types of non-sameness (similarity, diversity and opposition) on non-human animal learning in trial and reinforcement paradigms (Zentall 2021) or imprinting paradigms (Martinho and Kacelnik 2016)? A red big cube and a red small vertical parallelepipedon are examples of the different stimuli used by Martinho and Kacelnik (2016) to study imprinting in ducklings. The two shapes are of the same color, and they are aligned in front of the ducklings. They do not look very different to a human eye. But what if the stimuli represented other kinds of non-sameness? Would this reveal whether discrimination and relational learning follows global-to-local processing strategies or local-to-global attentive paths? (see De Lillo et al. 2005; Deruelle and Fagot 1998; Hodgetts et al. 2023; Hopkins and Washburn 2002).

Based on a review of animal studies, it has been recently put forward that sameness may be a natural concept that does not require learning (Zentall 2021). One interesting hypothesis is that this might also apply to opposition, especially considering that the ability to move in opposite directions in space might represent a significant variable in the avoidance behavior and the escape trajectories of animals (Domenici and Ruxton 2015; Kawabata et al. 2023). However, a robust basis of data is needed before a search for explanations can be carried out. We suggest that an interesting line to take would involve a new stream of research which re-thinks the dyadic same–different paradigm and operationalizes “different” in a triadic (i.e., similar, different, opposite) rather than a monolithic form.

## Figures and Tables

**Table 1:** Comparison between the ratings of opposition (O), difference (D) and similarity (S) associated with each of the 11 transformations (as according to the results from the Bonferroni post hoc tests relative to the interaction between Type of Relationship and Transformation). Columns I–III describe the transformation and the relative ratings; columns IV–VI show the results of Bonferroni post hoc tests, and columns VII–VIII summarize the outcome in terms of the difference between O and the other two relationships (D and S).

Transformations	Matched Ratings		Est	SE	z Ratio	O vs. D	O vs. S	O Different from Both S and D
Size	O	D	−0.514	0.146	−3.524	ns	O < S	
Size	O	S	−5.958	0.147	−40.512 ***			
Axis	O	D	1.439	0.148	9.702 ***	O > D	O < S	X
Axis	O	S	−5.211	0.147	−35.34 ***			
size + axis	O	D	−0.517	0.145	−3.573	ns	O < S	
size + axis	O	S	−3.451	0.146	−23.672 ***			
shape + size	O	D	−2.991	0.145	−20.585 ***	O < D	O < S	X
shape + size	O	S	−0.912	0.145	−6.288 ***			
Shape	O	D	−1.066	0.145	−7.361 ***	O < D	O < S	X
Shape	O	S	−1.592	0.145	−10.994 ***			
Direction	O	D	1.602	0.146	10.986 ***	O > D	O < S	X
Direction	O	S	−4.338	0.147	−29.493 ***			
shape+size+axis	O	D	−1.184	0.145	−8.183 ***	O < D	O > S	X
shape+size+axis	O	S	1.550	0.145	10.715 ***			
shape+axis	O	D	−1.304	0.145	−9.006 ***	O < D	O > S	X
shape+axis	O	S	1.135	0.145	7.850 ***			
size+direction	O	D	2.968	0.145	20.486 ***	O > D	O < S	X
size+direction	O	S	−0.776	0.145	−5.347 ***			
shape+direction	O	D	0.143	0.145	0.986	ns	O > S	
shape+direction	O	S	1.786	0.145	12.349 ***			
shape+size+direction	O	D	−0.579	0.145	−3.982 *	O < D	O > S	X
shape+size+direction	O	S	3.194	0.145	22.085 ***			

Note: * *p* < 0.05, *** *p* < 0.001, ns = non-significant *p*-value.

**Table 2:** Effect size of each transformation with respect to the identity pair. The 2nd–4th columns describe this in terms of the difference from the identity pair (a positive sign indicates that the transformation increases the Similarity, Diversity, or Opposition ratings with respect to the rating for the identity pair). The 5th–7th columns report Cohen’s d index of the effect size: (1) is used to indicate a large effect, (2) a medium effect and (3) a small effect. The z-ratio in the last three columns report the Bonferroni post hoc comparison test between the average rating for the transformation specified in the first column and that of the identity pair.

	EST (Mean Difference)			Cohen’s d (Effect Size)			z-Ratio (*p*-Value)		
Transformations	O	D	S	O	D	S	O	D	S
Size	−1.019	2.354	−2.145	−0.426(3)	0.984 (1)	−0.897(2)	−7.056 ***	16.251 ***	−14.588 ***
Axis	0.060	1.477	−1.811	0.025(3)	0.618(2)	−0.758(1)	0.417	9.986 ***	−12.229 ***
size+axis	0.278	3.654	−3.357	0.116(3)	1.528(1)	−1.404(2)	1.936	25.274 ***	−22.899 ***
shape+size	0.276	6.127	−5.899	0.116(3)	2.563(1)	−2.467(2)	1.916	42.363 ***	−40.538 ***
Shape	0.606	4.530	−4.890	0.253(3)	1.895(2)	−2.045(2)	4.214 *	31.364 ***	−33.605 ***
Direction	0.929	2.184	−1.818	0.389 (3)	0.914(1)	−0.760(2)	6.447 ***	15.051 ***	−12.334 ***
shape+size+axis	1.882	5.924	−6.756	0.787(3)	2.478(2)	−2.826(1)	13.101 ***	41.015 ***	−46.409 ***
shape+axis	2.264	6.426	−5.960	0.947(3)	2.688(1)	−2.493(2)	15.756 ***	44.452 ***	−40.954 ***
size+direction	2.895	2.784	−3.417	1.211(2)	1.165 (3)	−1.429(1)	20.148 ***	19.243 ***	−23.410 ***
shape+direction	3.032	5.748	−5.842	1.268(3)	2.404(2)	−2.443(1)	21.103 ***	39.793 ***	−40.146 ***
shape+size+direction	3.521	6.960	−6.762	1.472(3)	2.911(1)	−2.828(2)	24.503 ***	47.969 ***	−46.467 ***

Note: * *p* < 0.05, *** *p* < 0.001.

## Data Availability

The raw data set was deposited, under creative commons attribution license, in the IEEE data repository, which is available online at the following link: https://dx.doi.org/10.21227/hx8w-1813 (accessed on 31 July 2023).

## Notes

1 On the debate regarding whether there are qualitative differences between the underlying processes adopted by human and non-human species, see for instance (Carstensen and Frank 2021; Gentner et al. 2021; Katz and Wright 2021; Kroupin and Carey 2021; Smirnova et al. 2021; Pepperberg 2021).

2 There is an example of this in the structure-mapping theory of comparison (e.g., Markman and Gentner 1993). According to this model, the processing related to comparisons involves structural alignment and mapping between two representations. This facilitates the grasp of structural commonalities and thus also the application and extension of previously acquired knowledge to new instances (Gentner and Medina 1998).

3 An interesting exception was found by Medin et al. (1990) who analyzed the influence of relational qualities (e.g., the same color, or the right side smaller than the left side) and attributes (e.g., a triangular shape, or colored white) on people’s perception of similarity and diversity. They concluded that relational properties were more relevant when people were asked to judge similarities, while attributes were more relevant when they focused on differences (see also Markman 1996).

4 We have deliberately decided not to speak of 90° and 180° rotations since these would be geometrical rather than phenomenological descriptions. We wanted to keep the two types of changes in orientation distinct: (1) a change in the axis of orientation from vertical to horizontal and (2) a change in the direction or orientation applied while leaving the configuration within the same axis (i.e., upright-upside down with reference to the vertical axis).

5 In identity pairs, the only visible difference between the comparison figure and the standard figure concerned the localization of the two shapes (with the comparison figure to the right of the standard figure). We considered ratings given to these pairs as the baseline values for S, D and O associated with a mere replication of the same shape. The ratings given by participants to these pairs revealed that the difference in localization was irrelevant to the S judgments (i.e., the pair was rated as maximally similar) and the D judgments (i.e., the pair was rated as not different at all), while it was associated with a low O rating (the average rating for identity pairs 2.823). The latter result was due to the reciprocal position of the two shapes (one to the right, the other to the left), as the participants explained when asked specifically to comment on their response at the end of the experimental session.
